# Morgan County Medical Society

**Published:** 1868-01

**Authors:** John W. Craig

**Affiliations:** Secretary pro. tem.


### Proceedings of Societies.

### MORGAN COUNTY MEDICAL SOCIETY.

The Society held its regular monthly meeting in the office of the City Clerk, in Jacksonville, October 12, 1867.

The Society met pursuant to adjournment, at two o'clock P.M., the President, Dr. Henry Jones, in the chair.

Dr. C. T. Wilbur, Permanent Secretary, being absent, Dr. Craig, of Arcadia, was called to the desk.

The minutes of the last meeting were read and submitted for action.

It was suggested that the report was not explicit as to the *large* doses of calomel used in croup by Dr. H. K. Jones.

Dr. H. K. Jones criticised the minutes, objecting to some statements therein relative to his treatment of croup and diphtheria. Also to the Report of a case of epithelioma, by Dr. Prince, protesting that it was discourteous in Dr. P. to report the case, and that as the minutes stood he was left in a false position before the members of the Society and the community at large, inasmuch as the minutes failed to record his objections made at the time to the form of Dr. Prince's report; and suggesting that a revisory committee be appointed to examine the minutes prior to publication.

Dr. Edgar, of Jacksonville, remarked that in his opinion such a committee of censors was unnecessary; that his ideas of professional etiquette would allow Dr. Prince, as consulting surgeon, to report said case of epithelioma; defended the minutes as reported by the Secretary, and, as neither Drs. Prince or Wilbur were present, he moved to suspend further action on the minutes until the next meeting. The motion prevailed.

Moved, by Dr. Johnson, and carried, that hereafter the minutes of each meeting be approved by the Society before published.

Dr. Wm. S. Edgar proposed Dr. Allen, of Murraysville, for membership. Referred.

There being no further regular business before the Society, the special order of the day was taken up, and the deferred essay of Dr. Edgar was called for. The Doctor responded in a brief but telling article on the "General Principles of Diagnosis," full of facts, suggestive and practical. Stating that in the practice of medicine we recognize two great classes of indications, general and local. The former to be diagnosed chiefly from the history and sensations of the patient, while in the latter, lesions of structure may be detected from physical signs. That no examination of a patient could be considered complete which did not include, not only a history of the patient, but also of his family and ancestry. Especially in all important cases this should not be omitted, as we may here discover some idiosyncracy or proclivity to disease and death, which may clear up many an otherwise obscure and difficult case. Referred to Abernethy as the first of the profession to direct and fix attention to the connection of many local affections with constitutional derangements. Stating that the great achievement of medical diagnosis is to trace through the complex labarynth of reflex and direct nervous symtoms and sympathies, the true origin of the disease in question. Suggesting that our method of examination and final diagnosis will be greatly influenced by our general theory of disease, and more or less thorough knowledge of pathology, anatomy, and physiology; was sorry to acknowledge that some members of the profession are so preöccupied with theories of physiological and pathological changes incident to the nervous centres, together with the ebb and flow of the so-called vital principles as associated with intellectual and moral causes, that they readily account for all ailments, aches, and pains by some direct or reflex nervous trouble in the economy. With such, all protracted diseases are diagnosed typhoid fever, if fever attend, or neuralgia, if not attended by fever; as the former is understood by the community to be dangerous, and the latter endless, this diagnosis of course prepares the friends for the issue, be it good or evil. The Doctor did not understand this diagnosis to be always made as a matter of policy, but often as the consequence of the absence of a clear and definite opinion of the pathology of the case. But while we look away from physiology and human anatomy into fields of metaphysical theories and fancies, and follow entities of the imagination, no progress is made in diagnosis, however extended the practice or numerous the cases of observation may be. Much of this speculative pathology may originate in the natural tastes and habits of thought. Such minds more or less run after the vagaries of homœopathy, which removes practical medicine further and further from exact science. Observing, also, that the chief distinction between the scientific practitioner and empiric, is, that the former studies his case with reference to the pathology, while the latter groups symptoms and *prescribes*, trimming ingeniously, to hold his friends and make money.

Dr. C. Fisher, as previously announced, was, for want of time, unable to furnish an essay, and instead, reported two cases of dysentery conducted to a successful issue on the saline treatment. He took this occasion to report the above cases, since he had, on a previous occasion, discountenanced the practice, as suggested by his friend, Prof. Wilbur. The Doctor spoke at some length, eulogizing the practice.

Dr. Craig remarked that in the army he was generally successful with this plan of treatment; stating, however, that most of the cases were of recent attack, as in camp or on the march, where the men generally apply for treatment in the formative stage.

Dr. H. K. Jones thought it applicable in many cases of private practice; that where purgatives were indicated it was often the best, but thought close discrimination necessary here as elsewhere.

Dr. J. T. Cassell wished to know of those advocating the saline treatment, whether they ever knew of a recovery from true dysentery without *first* producing a biliary secretion, remarking that, in his opinion, no recovery could take place without it; that with a full, copious, and consistent flow of bile no case of simple dysentery could prove fatal; that mercury was not a *sine qua non* in producing a secretion of bile, many remedies being in successful use for this purpose, and that salts might so be used.

Dr. Henry Jones spoke at some length, reminding us of two forms of dysentery not generally referred to by writers on this subject, *i.e.*, the Summer, or Autumnal, and Winter varieties, wherein the symptoms were generally alike, but results strikingly different—the Winter variety seldom proving fatal, being of a simple catarrhal nature, and not attended by that hepatic derangement, as in the Autumnal. The Doctor related a recent remarkable case of dysentery as under his charge, not so much as to its therapeutical bearing as for its unusual symptoms, progress, etc., etc.

Dr. Edgar, Sr., remarked that he had considered the saline treatment indicated in congestion of the capillaries of the mucous membrane of the bowels. In the army it was valuable in the treatment of flux or diarrhœa, following exposure, lying on the cold, wet ground, etc., before they take to bed with symptoms of inflammation of the lower bowels, or dysentery proper.

Dr. Henry Jones, of Jacksonville, and Dr. Kimber, of Waverly, were appointed to write essays for next meeting.

With an expression of thanks to the gentlemanly City Clerk for the use of his rooms, the Society adjourned to meet at two o'clock P.M., on the second Thursday of November next.

JOHN W. CRAIG, *Secretary pro. tem.*

